# Assessment of Clinical Risk Management System in Hospitals: An Approach for Quality Improvement

**DOI:** 10.5539/gjhs.v7n5p294

**Published:** 2015-03-16

**Authors:** Jamileh Farokhzadian, Nahid Dehghan Nayeri, Fariba Borhani

**Affiliations:** 1 Department of Community Health Nursing, School of Nursing and Midwifery, Kerman University of Medical Sciences, Kerman, Iran; 2 Nursing and Midwifery Care Research Center, School of Nursing & Midwifery, Tehran University of Medical Sciences, Tehran, Iran; 3 Department of Medical Surgical Nursing, School of Nursing & Midwifery, Kerman University of Medical Sciences, Kerman, Iran

**Keywords:** clinical risk, clinical risk management, patient safety, quality improvement

## Abstract

**Background:**

Clinical risks have created major problems in healthcare system such as serious adverse effects on patient safety and enhancing the financial burden for the healthcare. Thus, clinical risk management (CRM) system has been introduced for improving the quality and safety of services to health care. The aim of this study was to assess the status of CRM in the hospitals.

**Methods:**

A cross-sectional study was conducted on 200 nursing staff from three teaching hospitals affiliated with the Kerman University of Medical Sciences in southeast of Iran. Data were collected from the participants using questionnaire and observational checklist in quality improvement offices and selected wards. The data were analyzed using SPSS version 20.

**Results:**

Almost, 57% of persons participated in at least one of training sessions on CRM. The status of CRM system was rated from weak to moderate (2.93±0.72- 3.18±0.66). Among the six domains of CRM system, the highest mean belonged to domain the monitoring of analysis, evaluation and risk control (3.18±0.72); the lowest mean belonged to domain the staff’s knowledge, recognition and understanding of CRM (2.93±0.66). There were no integrated electronic systems for recording and analyzing clinical risks and incidents in the hospitals.

**Conclusion:**

Attempts have been made to establish CRM through improvement quality approach such as clinical governance and accreditation, but not enough, however, health care should move toward quality improvement and safe practice through the effective integration of CRM in organizational process.

## 1. Introduction

At present, modern advances in health care, and changes in patients’ demographics have created significant challenges in health care practices. In this changing environment, serious deficiencies have been identified in the quality of care and patient safety (Briner, Kessler, Pfeiffer, Wehner, & Manser, 2010; Hickey, Forbes, & Greenfield, 2010). The high prevalence of clinical risks or health care risk, such as adverse events, near misses, errors, and other clinical incidents have created great concerns for healthcare organizations. Besides their effects on patients, they have significant socioeconomic impacts (Johnstone & Kanitsaki, 2006; Verbano & Turra, 2010).

The Institute of Medicine’s (IOM’s) report in 2000 (Kohn, Corrigan, & Donaldson, 2002) indicated that most of the clinical risks originated directly from defects and insufficiencies in the healthcare system (Adibi, Khalesi, Ravaghi, Jafari, & Jeddian, 2012). The systemic approach assumes that the system and its processes provide an environment leading to the occurrence of errors. Therefore, it was concluded that the system should supply a safety network and that efforts should be directed towards the management of risks (de Vries, Ramrattan, Smorenburg, Gouma, & Boermeester, 2008; Khalify-Nejad, Ateaei, & Hadizadeh, 2008). Since clinical risks can never be completely eliminated (Briner, Manser, & Kessler, 2013), systematic interventions should be implemented in order to be controlled and their effects are reduced to protect patients from risks (Verbano & Turra, 2010). To handle these challenges, one way is the establishment of a clinical risk management (CRM) system (Briner et al., 2013; Khalify-nejad et al., 2008).

The CRM system is defined as all instruments, structures, processes, and activities that focus on identifying, analyzing, preventing, monitoring, and managing clinical risks (Briner, Kessler et al. 2010). This approach would improve the quality of healthcare and help ensure the safety of patients, visitors, and employees, as well as reduce the costs associated with the healthcare system (Verbano & Turra, 2010).

In 2010, the Iranian Ministry of Health and Medical Education (MOHME) called for Clinical Governance (CG) as a framework for establishing excellence in clinical care (Dehnavieh et al., 2013; Sheikhtaheri, Sadoughi, Ahmadi, & Moghaddasi, 2013). The CRM is an essential component of CG (Webb et al., 2010). It plays a central role in the development and establishment of CG (Chiozza & Plebani, 2006). The healthcare system asked health providers to practice based on international standards of quality and patients’ safety (Mehrdad, Salsali, & Kazemnejad, 2008). Hospitals put special emphasis on educational programs in different aspects of CRM with the goal of educating all health care providers and culture building. The main objective of the CRM was to demonstrate the commitment of hospital management to monitoring clinical practices and protecting patients’ safety (Adibi et al., 2012).

Despite the emphasis of the World Health Organization (WHO) on the implementation of CRM (Dehghan, Dehghan, Sheikhrabori, Sadeghi, & Jalalian, 2013), there have been many indications that health care is not as safe as it should be and that patients’ rights are not properly respected (Johnstone & Kanitsaki, 2007). Studies have indicated that between 4% and 17% of patients suffer from some kind of harm as a result of clinical risks, including disability, morbidity, prolonged length of stay, and even death (Baker et al., 2004; Groves, Meisenbach, & Scott-Cawiezell, 2011; Hoonhout et al., 2009; Johnstone & Kanitsaki, 2007). Clinical risks have caused serious problems in healthcare and annually kill more people than AIDS or breast cancer (Adibi et al., 2012; Reynard & Stevenson, 2009). Besides the direct harm to patients, these risks create a significant financial burden for the healthcare system (Cho, Ketefian, Barkauskas, & Smith, 2003; de Vries et al., 2008). According to WHO, the probability of being harmed during medical treatment is much greater in developing countries, such as Iran (WHO, 2014). Iran does not have comprehensive reliable information on clinical risks (Bayazidi, Zarezadeh, Zamanzadeh, & Parvan, 2012; Sheikhtaheri et al., 2013).

As seen in other countries (Freeman & Walshe, 2004; Hogan, Basnett, & McKee, 2007; Huntington, Gillam, & Rosen, 2000; Marshall et al., 2002), previous research in Iran revealed despite great efforts in establishment of all components of CG particularly CRM in hospitals; some obstacles have been identified in the healthcare system, such as high workload, lack of financial and physical resources, organizational culture, insufficient training programs, inadequate participation in education (Adibi et al., 2012), rapid change of managers, inefficient leadership support, and inadequate assessment and monitoring of the efficacy of CRM (Dehnavieh et al., 2013).

The study indicated that CRM was in the very early stages of development and that Iran’s health system is still far away to achieve international standards (Davoodi et al., 2014). There are no integrated systems for recording, reporting, and analyzing clinical incidents, e.g., Patient Safety Information Systems (Sheikhtaheri et al., 2013). The results of another study demonstrated that a hospital in Tehran have achieved only moderate results in implementing the various elements of CRM. Researchers have proposed that hospitals require continuous evaluation of CRM (Zaboli, Karamali, Salem, & Rafati, 2011). Despite the multitude of programs, no comprehensive study investigated and assessed the status of CRM in teaching hospitals. Therefore, to develop CRM and an appropriate strategy for achieving quality improvement and also monitoring progress, hospitals must acquire systematic information concerning their strengths and weaknesses.

Since the implementation of healthcare policies is centralized in Iran and there is no difference among provinces (Dehghan et al., 2013), therefore the aim of this study was to assess the status of CRM system in teaching hospitals in Kerman, the main city in southeast of Iran.

## 2. Materials and Methods

### 2.1 Research Design and Setting

This cross-sectional study was conducted in 2014 at three teaching hospitals affiliated with the Kerman University of Medical Sciences. Kerman is the largest city in southeastern Iran, with a population of 534, 441, and CRM system was being actively implemented in hospitals.

### 2.2 Data Collection

The study population consisted of nursing staff (nurses, healthcare workers and nurses’ managers) who were employed at the time of data collection (n=820) in the hospitals. The sample size (n=200) was calculated based on the sample size formula of Cochran, where α=0.05, d=0.06, and σ=0.05.

One researcher collected the data. Interviews were used to complete the questionnaires and checklists. Observational data were collected from selected wards and from documentation in the Office of Quality Improvement. The quota sampling technique was used (based on the proportion of clinical staff at each hospital). Thus 56, 67, and 77 (200 participants) were enrolled from the three hospitals. Given that the CRM programs were running systematically and integrated in all wards of the hospitals, we selected the different wards i.e., Cardiac Intensive Care Unit (CCU), Intensive Care Unit (ICU), medical and surgical wards, emergency and management. Then, 148 nurses, 30 healthcare workers, and 22 nurses’ managers (matron, clinical and educational supervisors, and head nurse) participated from these wards. Staff members with work experience more than one year were considered to be eligible for the study.

### 2.3 Measurement Tools

Two tools were used for data collection, i.e., a structured observational checklist and a questionnaire. The checklist was developed by the researchers based on the literature (Mostafa, 2009; Zarezade et al., 2013). It included 25 items to observe the present programs and measurements for developing CRM system, such as incident reporting, and other various aspects of CRM (Table 4).

**Table 1:** Demographic information (n=200)

		Frequency	Percentage
Sex	Female	160	80.0
	Male	40	20.0

Age groups	<30	48	24.0
	30-35	63	31.0
	36-40	54	27.5
	>40	35	17.5

Experience (Year)	<5	47	23.5
	5 – 10	36	18.0
	11-15	41	20.5
	16-20	37	18.5
	>20	39	19.5

	Master of nursing	6	3.0
	Bachelor of nursing	156	78.0
	Associate	8	4.0
	Diploma	30	15.0

Job title	Nurse-managers	22	11.0
	Nurse	148	74.0
	Healthcare worker	30	15.0

Work shift	Rotation	53	26.5
	Fixed	147	73,5

Attending training	Yes	114	57.0
	No	86	43.0

**Table 2:** Mean scores of each of the domains of CRM

Domains of CRM	Mean score*	SD
1. Staff’s knowledge and recognition of CRM	2.93	0.72
2. Status of CRM organizing	3.01	0.68
3. Status of policies and procedures of CRM	3.06	0. 67
4. Status of CRM training	3.12	0.73
5. Position of CRM	3.14	0.57
6. Monitoringof analysis, evaluation and risk control	3.18	0.66

* Mean scores calculated from 1 (very low) to 5 (very high).

**Table 3:** Comparison of mean scores in each domain with demographic variables

	Experience		Work shift		Attending training	

	f	P	T	P	T	P
1. Staff’s knowledge and recognition of CRM	2.40	0.038	2.63	0.010	5.06	0.000
2. CRM organizing	3. 94	0.002	2.20	0.030	4.49	0.000
3. Policies and procedures of CRM	2.87	0.016	2.33	0.022	3.80	0.000
4. CRM training	4.23	0.001	2.60	0.010	4.30	0.000
5. Position of CRM	3.10	0.010	2.10	0.040	4.21	0.000
6. Monitoring of analysis, evaluation and risk control	3.50	0.004	2.55	0.012	3,36	0.001

**Table 4:** Assessment of study settings for the presence of programs of CRM

Items	Hospital a	Hospital b	Hospital c
Risk management committee and team	✓	✓	✓
Quality improvement committee, team and office	✓	✓	✓
Patient safety committee and team	✓	✓	✓
Staff and patient safety officer	✓	✓	✓
The risk manager	✓	✓	✓
Educational program & culture building	✓	✓	✓
Patients’ complaints management	✓	✓	✓
Program of nosocomial infections control	✓	✓	✓
Reporting system for adverse drug reactions (ADRs)	✓	✓	✓
Safety action plan	✓	✓	✓
Complications of blood and blood products form	✓	✓	✓
Licensure and accreditation bodies	✓	✓	✓
Incident reporting form	✓	✓	✓
Incident reporting not book	✓	-	-
Online and web-based reporting form	✓	-	✓
Incident report form in patient file	-	-	-
Patient safety information systems (PSIS)	-	-	-
Patient safety reporting system (PSRS)	-	-	-
Reviews incident reports weekly	✓	✓	✓
Root-cause analysis of sentinel event	✓	✓	✓
Failure mode and effects analysis (FMEA)	✓	✓	✓
Medication administration cart	✓	✓	✓
Labeling of medication packages	✓	✓	✓
System of individual distribution of medication	✓	✓	✓
Electronic system for medication request	✓	✓	✓

The questionnaire was developed by a team that was comprised of faculty members in “the Department of Health Care Services Management at Baqiyatallah University of in Tehran”, and permission was acquired for its use (Zaboli et al., 2011). The fundamental patterns such as the occupational health and safety management system (OHSAS), ISO 18000 standards, recommendations of Joint Commission Accreditation of the Health Institutions (JCAHO), literature review, and the authorities’ and researchers’ opinions were applied to design the questionnaire.

The questionnaire was divided into seven sections, i.e., 1) socio-demographic data, such as gender, age, work position, work shift (fixed, rotation), degree, experience, and attendance at CRM training courses; 2) staff’s knowledge, understanding and recognition of CRM (eight questions); 3) the status of organizing the CRM (seven questions); 4) the policies and procedures (seven questions); 5) evaluation of the status of CRM training (eight questions); 6) the position of CRM in the hospitals (six questions); and 7) the status of monitoring, analysis, evaluation, and risk control (nine questions). The questionnaire was developed based on the Likert 5-point scale (very low, low, medium, high, and very high).

The content validity was obtained by experts’ opinions, and the experts consisted of nurses’ managers and risk managers from the hospitals, a healthcare risk consultant, and the faculty of nursing.

The reliability was assessed using Cronbach’s alpha coefficient (α= 0.85). This checklist was based on “yes” or “no;” answers and no grade scores were used, so validation was not required. In addition, Zaboli et al assessed validity and reliability of their questionnaire using the content validity and test retest validity (coefficient r=83%).

### 2.4 Statistical Analysis

The data were analyzed using SPSS IBM version 20. Descriptive statistics with frequency and percentage, mean, standard deviation (SD), and analytical statistics were performed to study the differences between socio-demographic groups and the mean scores of CRM variables using independent samples *t*-test and analysis of variance (ANOVA).

### 2.5 Ethical Considerations

The study was conducted as one part of the first author’s PhD dissertation. This study proposal was approved by the research ethics committee of Kerman University of Medical Sciences (approval number: K/93/145). Ethical issues (Including plagiarism, Informed Consent, misconduct, data fabrication and/or falsification, double publication and/or submission, redundancy, etc) have been completely observed by the authors.

## 3. Results

### 3.1 Demographic Information

The sample was comprised of 200 respondents. The majority of them was female (80.0%), and the respondents’ ages were between 30 and 35 (31.0%). About 23.5% of the participants had less than five years’ experience, and 74.0% were nurses (Table 1).

### 3.2 The Domains of CRM

Table 2 shows the mean scores of six domains of CRM. Generally, among all six the domains of CRM, the highest mean score belonged to the status of monitoring of analysis, evaluation and risk control (3.18±0.66), and the lowest score belonged to staff’s knowledge, understanding and recognition of CRM (2.93±0.72).

The mean score of six domains of CRM based on gender, age group, degree, and job title did not have any statistically significant difference (P<0.05). There was a statistically significant difference among the mean score of the CRM domains with experience, work shift, and attending training of CRM (Table 3).

### 3.3 Observational Data

Table 4 describes the programs of CRM that were present in the study hospitals. Almost all of study settings were implementing most measures of CRM. Despite these initiatives, there were no incident report forms in patients’ files; electronic systems, such as Patient Safety Information Systems (PSIS); or the Patient Safety Reporting System (PSRS).

## 4. Discussion

This study assessed the status of CRM in six domains in teaching hospitals, i.e., 1) staff’s knowledge, understanding and recognition of CRM, 2) the status of CRM organizing, 3) the status of policies and procedures of CRM, 4) status of the CRM training, 5) CRM position, and 6) monitoring of analysis, evaluation, and risk control. The findings indicated that implementing and developing domains of CRM ranged from poor to moderate.

In our study, 57% persons participated in at least one of the specialized training sessions on CRM. This result was similar to that of the study done on clinical governance (Dehghan et al., 2013). It is possible that the lack of attendance at the training sessions related to inappropriate planning times, such as planning in the morning shift, concurrency with work shifts, high workloads, and little motivation to participate

In present study, the poor to moderat status of the CRM in six domains may reflect the fact that CRM and patients’ safety are new programs in Iran, and the healthcare system has yet to resolve the problems in the current situation; also the current safety culture is not appropriate. These findings are consistent with other studies (Davoodi et al., 2013; Johnstone & Kanitsaki, 2006; Mostafa, 2009; Zaboli et al., 2011; Zarezade et al., 2013). These studies indicated that the status of CRM was not satisfactory in the study settings and that the participants had low knowledge about the CRMS concept and the general criteria of incident reporting.

The results showed that the staff’s knowledge, understanding and recognition of CRM was poor. Researchers in a study showed that low knowledge regarding concepts and elements of CRM resulted in weak participation in the programs of CRM, such as reporting and analysis. Thus, hospital managers should focus on learning different aspects of CRM, encouraging all healthcare providers to do the same, and building a culture in which patients’ safety is a top priority (Adibi et al., 2012). Recognition of risk is a critical stage in the development of CRMS, and it depends on maintaining a culture of honesty, trust, integrity, and open communication among patients, families, and healthcare providers (Neale, 1997).

According to our results, the status of CRM organizing, the management policies and procedures, and CRMS training was moderate. It may be evident that educational programs and activities of mangers regarding the preparation and availability of the regulations and rules of CRM are in very low level in hospital contexts. The CRM must be implemented as strategies and action plans, and clinical staff must be trained as well so that they are fully aware of and supportive of the different CRM domains, principles, structures, guidelines, and schemes (Adibi et al., 2012; Handel & McConnell, 2007; Neale, 1997; Neale, Woloshynowych, & Vincent, 2001; Vaismoradi, 2012). Our findings were similar to another study conducted in Iran in which there was no compliance with CRM requirements in the different wards of the hospital (Yarahmadi, 2009).

An action research study in Italy explained that graduate nurses still have not acquired pertinent information about CRM processes in health care. The main barriers were an inadequate educational curriculum and the lack of a systematic, focused, and uniform CRM education program for the development of the graduates’ capabilities in CRM (Johnstone & Kanitsaki, 2006). The same situation exists among the nursing curricula in Iran. Therefore, it is important to investigate how to design the nursing curriculum in order to include the different aspects of CRM and patients’ safety into the educational curriculum. Moreover, future studies can help educators facilitate the process of the transfer of CRM knowledge into practice (Vaismoradi, Bondas, Jasper, & Turunen, 2012b)

The findings on the domain related to the position of CRM highlighted that the creation of appropriate knowledge and attitudes is necessary but not sufficient. The staff may still encounter barriers, such as social and organizational factors that include the lack of resources; the lack of facilities and equipment; insufficient staff; demanding workloads; lack of support from leaders; and the weakness of teamwork.

In this study, the status of monitoring of risk control, analysis and evaluation in the hospitals was at a moderate level. Insufficient participation in processes may be because of culture blaming, poor feedback to staff, resistance of managers and physicians, high turnover of clinical staff, and limited resources in Iran’s hospitals. Several studies have evaluated how to manage the risk in hospitals using the Failure Modes and Effects Analysis (FMEA) method. These studies depicted FMEA as an efficient and effective technique for identifying and prioritizing the improvable points of work processes. It can enhance staff precision and attract their attention to their possible professional weaknesses in CRM (Attar Jannesar, Tofighi, Hafezimoghadam, Maleki, & Goharinezhad, 2010; Rahimi, Kharazmi, & Shafeghat, 2013; Sedaghat, Ghanjal, Delavari, & Tavakoli, 2008). Generally, we inferred that the implementation of CRM will require to focus on the needs of patients and the needs of health care providers. CRM can be promoted by multifaceted interventions, such as human, financial, time, clinical, and technical resources, as well the application of information technology, the use of information, and building a culture that is focused on patients’ safety. The organizational leaders must facilitate this process by creating necessary institutional infrastructures and strategic planning, management systems, and reinforcement of teamwork and professional communication to build positive attitudes among care providers towards strategies accreditation and clinical governance. In this regard, they must utilize leadership and management theories such as transformational leadership, participative, and change management.

The findings revealed significant difference between the mean scores of research domains of CRM based on experience, work shift, and attending training. These staff members may be more involved in the CRM program since usually staff with more experience work in morning shift and take greater responsibility for the implementation of CRM programs. Zaboli et al. reported the absence of a significant difference between domains of CRM and sociodemographic variables (Zaboli et al., 2011). This difference may be related to sample size and the backgrounds of the participants.

The observational data revealed that all of the study settings were implementing programs, instruments, measurements, and procedures for developing CRM. However, there were no national electronic systems, such as Patient Safety Information Systems (PSIS), for recording and analyzing incidents. There are two national safety programs, i.e., the control of nosocomial infections and a reporting system for adverse drug reactions (ADRs). This result is consistent with the findings of Sheikhtaheri et al., which suggested that Iranian hospitals need to critically develop systems such as PSIS for developing CRM. Health researchers should design standard forms for reporting, standardized data sets, technique for data analysis, and feedback. Establishing such systems is an opportunity to ensure data quality and prevent problems, such as incomplete or duplicate reports, data inconsistency, and missing important information. It disseminates lessons learned between healthcare organizations and knowledge shared among professionals (Sheikhtaheri et al., 2013)

This study was limited by the assessment of the status of CRM only in three teaching and physical care hospitals affiliated with the Kerman University of Medical Sciences. A more comprehensive profile and insight into the CRM require inclusion of hospitals of different types, such as psychiatric, ownership, geographical status, and cultural and environmental conditions. Such a survey can lead to national strategies to improve the CRM and identify challenges that may not have been captured by the limited scope of this study.

## 5. Conclusions

The findings of this research indicated that, despite the establishment of clinical governance and accreditation approaches, the status of CRM is not appropriate. Health care in southeast Iran is not moving enough towards high quality and safe practice, and it is still away from international standards. The findings of this study will be useful for hospitals and nursing leadership and management, risk managers, policy-makers, decision-makers, and medical educators in Iran to develope an appropriate strategy for achieving quality improvement, promoting CRM, and planning training programs. This assessment provided systematic and comprehensive information for developing appropriate interventions to integrate effective CRM system in health care. We suggest additional quantitative and qualitative research to assess and continuously monitor the elements of CRM and to compare and exchange information among the various hospitals. Future research can promote learning and sharing of high-quality, safe practices. In addition, we suggest that researchers create a standardized scale for assessing the key element of CRM for use by health care providers.
